# Porous scaffolds with the structure of an interpenetrating polymer network made by gelatin methacrylated nanoparticle-stabilized high internal phase emulsion polymerization targeted for tissue engineering†

**DOI:** 10.1039/d1ra03333f

**Published:** 2021-06-25

**Authors:** Atefeh Safaei-Yaraziz, Shiva Akbari-Birgani, Nasser Nikfarjam

**Affiliations:** Department of Chemistry, Institute for Advanced Studies in Basic Sciences (IASBS) Zanjan 45137-66731 Iran nikfarjam@iasbs.ac.ir +982433153232 +982433153132; Department of Biological Sciences, Institute for Advanced Studies in Basic Sciences (IASBS) Zanjan 45137-66731 Iran

## Abstract

The interlacing of biopolymers and synthetic polymers is a promising strategy to fabricate hydrogel-based tissue scaffolds to biomimic a natural extracellular matrix for cell growth. Herein, open-cellular macroporous 3D scaffolds with a semi-interpenetrating network were fabricated through high internal phase emulsion templating. The scaffolds are prepared by (I) the curing of PEG diacrylate (PEGDAC) and gelatin methacrylate (GelMA) in the continuous aquatic phase of a coconut oil-in-water emulsion stabilized by GelMA nanoparticles, and (II) the removal of the internal phase. The effect of the main contributing parameters such as pH, GelMA content, and GelMA/PEGDAC weight ratio on the emulsion features was investigated systematically. Due to the isoelectric point of GelMA at around pH 6, the GelMA particle (aggregation) size decreased at both sides of pH from 1000 to 100–140 nm because of the increased number of positive and negative charges on GelMA. These GelMA nanoparticles were able to produce stable emulsions with narrowly distributed small emulsion droplets. Moreover, the stability and emulsion droplet size were enhanced and increased, respectively, with GelMA content increasing and GelMA/PEGDAC weight ratio decreasing. These trends lie in the prevented coalescence phenomenon caused by the improved viscosity and likely partially formed network by GelMA chains in the continuous phase. Hence, the following formulation was selected for scaffold preparation: *φ*_oil_ = 74%, pH = 12, GeMA = 4 wt%, and GelMA/PEGDAC = 10/8. Then, PCL in different contents was infiltrated into the scaffold to balance hydrophilicity and hydrophobicity. The cell culture assay proved that the scaffold with a pore size of 60–180 μm and containing 51.2 wt% GelMA, 10.3 wt% PEG, and PCL 27.2 wt% provided a suitable microenvironment for mouse fibroblast cell (L929) adhesion, growth, and spreading. These results show that this strategy suggests promising culture for tissue engineering applications.

## Abstract 2

The interlacing of biopolymers and synthetic polymers is a promising strategy to fabricate hydrogel-based tissue scaffolds to biomimic a natural extracellular matrix for cell growth.

## Graphical abstract

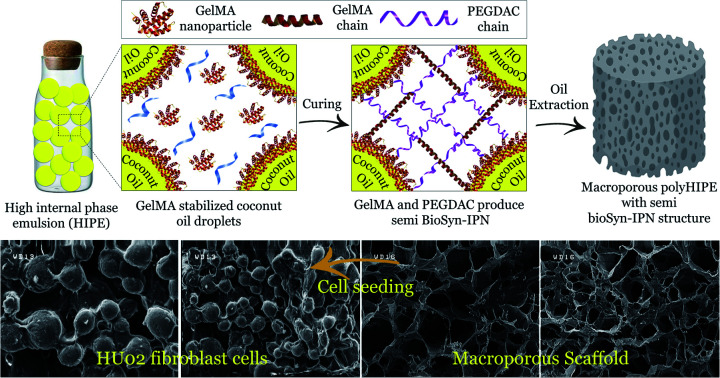

## 1. Introduction

The emerging engineering field of biomimetic extracellular matrices (ECM) is crucial in regenerative medicine and tissue engineering. The hierarchical network architecture of native ECM provides mechanical support and a biological interactive microenvironment for cellular and organ fusion. The interpenetrated network (IPN) structure of native ECM consists of crosslinked proteins interlocked with biomacromolecules. The increased demand for replication of ECM has led to developing physically or chemically crosslinked hydrogels as popular scaffold platforms for 3D cell culturing.^1^ The engineered hydrogels for ECM replacement must be fabricated under cytocompatible conditions. They should biomimic the native ECM attributes to achieve the precise biological microenvironment for a particular cell type culturing. The dimensional stability, high water uptake, and optimal transport of nutrients and oxygen are favorable features of hydrogels for cell culture aspects. A wide variety of synthetic and biological macromolecules have been developed for this kind of hydrogel, such as poly(vinyl alcohol), poly(ethylene glycol), alginate, chitosan, fibrin, gelatin, chondroitin sulfate, hyaluronic acid, and agarose.^2,3^ Although the synthetic macromolecules are structurally homogenous, nontoxic, and amenable to mechanical tuning, they intrinsically lack biological activity with limited proliferation, migration, and organization of cells. In contrast, the biomacromolecules, especially those derived from native ECM, have bioactivity with excellent cell proliferation, migration, and differentiation. Nonetheless, they have structural heterogeneity and suffer from mechanical weaknesses.^4^ Accordingly, the integration of synthetic and biological macromolecules can be a good way to design an ideal hydrogel for ECM engineering.^5,6^ Interlacing the biological and synthetic macromolecules in the IPN structure resulted in BioSyn–IPN, which can exhibit higher water uptake, high strength, and bioactivity such as the native ECM.^1,6–9^ Among synthetic polymers, poly(ethylene glycol) (PEG) is very popular to make biological hydrogels due to its unique features such as non-immunogenicity, non-toxicity, favorability to oxygen and nutrient transport, mechanical strength, and it can fabricate hydrogels under cytocompatible conditions.^10,11^

Gelatin (a natural polymer derived from controlled hydrolysis of the triple-helix structure of collagen into single strain molecules) and its modified products such as gelatin methacrylate (GelMA) retain the same bioactivity of collagen. RGD binding sequences of GelMA allow cells to bind directly to the hydrogels made by GelMA and cells can enzymatically remodel and degrade the GelMA hydrogels.^12–14^ Several methods have been developed to fabricate macroporous hydrogels for biomedical applications, such as 3D bioprinting, incorporating biocompatible porogens, electrospinning, photo-patterning, microfluidic assisted systems, stereolithography, sacrificial bio-printing, freeze-drying, *etc.*^15–17^ But among these methods, emulsion templating has been attractive due to its convenient capability to control morphology, porosity, surface area, pore size, and physical properties by changing the parameters with no need for any expensive equipment. High internal phase emulsions (HIPE) with a dispersed phase volume fraction of at least 74% (maximum packing fraction of monodispersed spherical emulsion droplets) are commonly used to manufacture macroporous polymeric materials. The emulsion droplets can be exploited to create macropores in a monolithic solid structure if the monomers or macromers are polymerized in the HIPE continuous phase followed by extraction of the droplet phase. This process leads to an interconnected structure with a micrometric pore size, commonly known as polyHIPE. In polyHIPEs, the pores are separated from neighbors by only an extremely thin perforated polymer film.^18,19^ These highly interconnected structures with mechanical integrity could provide available space for cell attachment and migration as well as nutrient transportation to cells and removal of produced wastes by necrotic regions within the scaffold.^20,21^ Unlike conventional polyHIPEs (stabilized by the high content of harmful surfactants^22^) with low permeability due to the small pore and pore throat sizes,^23^ the polyHIPEs stabilized by solid particles, known as Pickering polyHIPEs, are more attractive in bio-related applications especially scaffold fabrication for tissue engineering.^24,25^ This is mostly because of the low demand for stabilizers during the fabrication process, defined hierarchical structures, and high macroporosity. In the Pickering HIPE, solid particulate stabilizers are irreversibly adsorbed on or self-assembled at the oil–water interface to create a mechanically robust layer around the droplet to hinder droplet coalescence, creaming and Ostwald ripening.^26^ The Pickering polyHIPEs made by nontoxic solid particles have gained more attention over recent years to fabricate scaffolds for tissue engineering and regenerative medicine.

Since gelatin is naturally amphiphilic and can easily assemble into aggregates in different degrees under the specific processing conditions of pH and temperature, it has been highly desired for stabilizing HIPEs for biomedical applications.^27–29^ Because of gelatin particles' softness, they can likely deform and occupy large oil–water interface areas to form a viscoelastic layer around droplets, leading to enhanced emulsion stability. Hence, this report's main aim is to demonstrate the fabrication of porous hydrogel scaffolds with a semi BioSyn–IPN structure through Pickering emulsion templating. The related coconut oil-in-water (o/w) Pickering HIPEs were stabilized using approximately monodispersed GelMA nanoparticles as the sole stabilizer. The continuous aquatic phase contained a defined ratio of GelMA and PEG diacrylate (PEGDAC). The hierarchical porous hydrogel structure was obtained after crosslinking of macromers followed by the removal of coconut oil. The effect of contributing parameters on emulsion features such as pH, particle content, and GelMA/PEGDAC weight ratio was examined precisely to find an optimized formulation for emulsions with a uniform size distribution. Finally, the potential applicability of the ensuing soft-gelled porous structure as a scaffold in tissue engineering in the absence and presence of polycaprolactone at different ratios was assessed.

## 2. Experimental

### 2.1. Materials

Gelatin from porcine skin, poly(ε-caprolactone) (PCL with *M̄*_n_ = 80 000 g mol^−1^), a dialysis bag with a cut-off of 12 kDa, Hoechst dye and 3-[4,5-dimethylthiazol-2-yl]-2,5-diphenyl tetrazolium bromide (MTT powder) were purchased from Sigma-Aldrich. Poly(ethylene glycol) (PEG, *M̄*_n_ = 1000 g mol^−1^), acryloyl chloride, methacrylic anhydride, sodium hydroxide, diethyl ether, ethanol, and DMSO were obtained from Merck. Ammonium persulfate (APS), potassium dihydrogen phosphate (KH_2_PO_4_), and ethylenediaminetetraacetic acid (EDTA) were prepared from Fluka. Toluene and acetone were provided by Dr Mojallali Co. Coconut oil as the internal phase was obtained from the market. Distilled water was freshly used for all the experiments.

### 2.2. Preparation of macromers

#### Synthesis of gelatin methacrylate (GelMA)

GelMA was prepared by the freeze-drying treatment method.^30^ In detail, 3 g gelatin was completely dissolved in 50 ml phosphate buffer (pH 7.5) at 50 °C for 1 h. Then, methacrylic anhydride (MA) (2.4 ml, 16 mmol) was added dropwise while vigorously stirring for 3 h. After complete reaction, the mixture was diluted with 100 ml PBS and dialyzed against deionized water at 40 °C for 72 h to remove the unreacted MA and other impurities. The resulting GelMA was obtained as a white solid after lyophilization.

#### Synthesis of poly(ethylene glycol) diacrylate (PEGDAC)

For acrylation of PEG, 3 g PEG with a molecular weight of 1000 g mol^−1^ were dissolved in 15 ml of dry tetrahydrofuran (THF) while vigorously stirring in an ice-water bath under an argon atmosphere. Dried triethylamine (1.3 ml) was added dropwise to the PEG solution at 0–5 °C. Then acryloyl chloride (0.6 ml, 7 mmol) was added dropwise to the solution and stirred overnight under an argon atmosphere. The formed triethylammonium chloride salt was filtered, and the PEG diacrylate (PEGDAC) product was precipitated in cold diethyl ether. For further purification, the PEGDAC was dissolved in THF and precipitated in diethyl ether another two times.^31^ The final PEGDAC was dried in a vacuum oven at 50 °C for 24 h.

### 2.3. Preparation of o/w Pickering HIPE

A defined amount of GelMA and PEGDAC macromers was added to the distilled water and stirred vigorously at 40–50 °C for an hour, and then the pH of the solution was adjusted using NaOH and HCl (4 M). Then, coconut oil as an internal phase was added to the aqueous solution to reach a volume fraction (*φ*) of 74% and sonicated for 20 s using a Qsonica-Q700, 700 W, 20 kHz with an amplitude of 20% to prepare the coconut oil-in-water HIPEs. To precisely assess the effect of contributing parameters such as pH, GelMA content, and GelMA/PEGDAC weight ratio on the emulsion features, three series of HIPEs were prepared according to Table 1. The morphology of emulsion droplets was observed immediately by optical microscopy (Nikon E200) equipped with a CCD camera (TrueChrome Metrics, China). The size distribution of emulsion droplets was obtained by coding in Matlab software (see ESI†), and the following equation calculated the volume-surface mean droplet diameter (*d*_3,2_): 
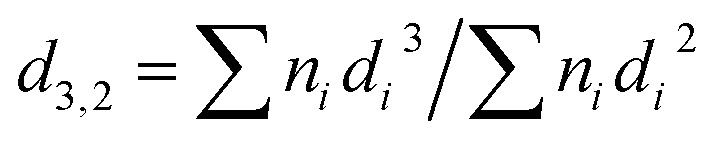
, where *n*_*i*_ is the number of droplets with a diameter of *d*_*i*_.

**Table 1:** The formulation of the coconut oil-in-water (o/w) Pickering HIPE stabilized by GelMA nanoparticles. The volume fraction of coconut oil (*φ*_oil_ = 74%) as the internal phase was kept constant for all samples

Series	Sample	GelMA^a^, wt%	PEGDAC^a^, wt%	GelMA/PEGDAC weight ratio	pH
pH	HIPE-pH = 2	1	0.1	10:1	2
	HIPE-pH = 4	1	0.1	10:1	4
	HIPE-pH = 6	1	0.1	10:1	6
	HIPE-pH = 8	1	0.1	10:1	8
	HIPE-pH = 10	1	0.1	10:1	10
	HIPE-pH = 12	1	0.1	10:1	12
GelMA nanoparticle	HIPE-GelMA-0.5	0.5	0.05	10:1	12
	HIPE-GelMA-1	1	0.1	10:1	12
	HIPE-GelMA-1.5	1.5	0.15	10:1	12
	HIPE-GelMA-2	2	0.2	10:1	12
	HIPE-GelMA-3	3	0.3	10:1	12
	HIPE-GelMA-4	4	0.4	10:1	12
GelMA/PEGDAC	HIPE-GelMA : PEGDAC-1 : 0.2	4	0.8	10:2	12
	HIPE-GelMA : PEGDAC-1 : 0.4	4	1.6	10:4	12
	HIPE-GelMA : PEGDAC-1 : 0.6	4	2.4	10:6	12
	HIPE-GelMA : PEGDAC-1 : 0.8	4	3.2	10:8	12
	HIPE-GelMA : PEGDAC-1 : 1	4	4	10:10	12

a Weight percent related to the continuous aquatic phase.

### 2.4. Fabrication of porous structures *via* Pickering HIPE polymerization

Taking cues from the nature of GelMA to create aggregates in the aqueous phase under different conditions, the GelMA aggregates were used in an excess amount to stabilize emulsion oil droplets and be cured along with PEGDAC in the continuous aquatic phase. After precisely investigating the emulsion features based on the droplet size distribution and stability over time, the following optimized recipe was formulated for polymerization the same as the method mentioned above: *φ*_oil_ = 74%, pH = 12, GelMA content of 4 wt%, PEGDAC content of 3.2 wt%, GelMA/PEGDAC weight ratio and APS content of 7 wt% related to both macromers' total weight. The prepared HIPEs were quickly transferred into the plastic cylindrical mold with a dimension of 3 × 5 cm and polymerized in an oven at 70 °C for 24 h. Next, the gelled materials' internal phase was removed using a Soxhlet using toluene for 24 h with a cycling time of 20 min. Afterward, the unreacted macromers and other impurities were washed out using a Soxhlet with water for 24 h. Finally, the ensuing porous Pickering polyHIPE was lyophilized using a freeze-dryer and called PolyHIPE–GelPEG. To improve the prepared porous structure's dimensional stability, ethylene glycol dimethacrylate (EGDMA) was added to the Pickering HIPE's aqueous phase in three concentrations of 0.09, 0.2, and 0.27 M and then polymerized and extracted with toluene and water the same as the procedure mentioned above. The prepared final product was lyophilized using a freeze-dryer and called X-PolyHIPE–GelPEG.

### 2.5. Pickering polyHIPE incorporated with PCL as a final scaffold

To render hydrophobicity to the porous Pickering polyHIPE, the PCL in different contents was introduced inside the porous structure through the polymer solution infiltration method. For this purpose, the obtained porous Pickering polyHIPEs were added to the PCL/toluene solutions in different concentrations of 2.5, 5, 7.5, and 10 wt%. Then, the PCL chain diffusion into the porous structures was performed under controlled vacuum pressure for 10 min. Under the vacuum, the polymer solution was replaced by trapped air inside the porous structures and precipitated on the pores' wall existing in the porous structures during the drying process. Then, the final scaffolds were dried in a vacuum oven at 60 °C for 24 h and called X-PolyHIPE–GelPEG–PCL-0, X-PolyHIPE–GelPEG–PCL-2.5, X-PolyHIPE–GelPEG–PCL-5, X-PolyHIPE–GelPEG–PCL-7.5, and X-PolyHIPE–GelPEG–PCL-10 in which the last number of nomenclatures shows the used PCL concentration.

### 2.6. 3D cell culture

*In vitro* cell culture assay was performed to evaluate the potential use of the prepared scaffolds for tissue engineering. Therefore, the scaffolds with different ratios of PCL, *i.e.*, X-PolyHIPE–GelPEG–PCLs, were cut into slices with a thickness of ∼5 μm and were sterilized by soaking in 70% ethanol for 24 h. After washing with PBS several times, the scaffolds were put into a 96-well plate. 2 × 10^4^ mouse fibroblast cells (L929) were seeded on the scaffolds and 1 ml of culture medium (RPMI 1640 containing 10% fetal bovine serum, 1% penicillin, and streptomycin) was added to the cells and incubated in a CO_2_ incubator at 37 °C for 48 h. The cell viability on the scaffold was evaluated using a standard protocol of MTT assay. Briefly, after replacing the culture medium with a fresh medium, 10 μl of MTT working solution with a concentration of 5 mg ml^−1^ was added to each well, and the plate was incubated at 37 °C for 4 h. Then after aspiration of the medium, 100 μl of DMSO was added to each well and the plate was incubated at 37 °C for another 1 h. The absorbance was measured at 540 nm using an ELISA plate reader (BioTek Instruments, USA). The MTT assay was performed in triplicate.

### 2.7. Characterization

#### Fourier-transform infrared spectroscopy (FT-IR)

The native gelatin, GelMA, PEG, PEGDAC, and PolyHIPE–GelPEG–PCLs were characterized using FT-IR spectroscopy (Bruker vector 22 spectrophotometer, Germany) by preparing their KBr pellets from 400 to 4000^−1^.

#### Dynamic light scattering (DLS) and zeta potential

Size, size distribution, and zeta potential of GelMA nanoparticles at different pH values were measured by dynamic light scattering (DLS) using a Nano ZS ZEN 3600 (Malvern, UK). All measurements were performed at a wavelength of 633 nm at 25 °C. The GelMA dispersion was diluted to an appropriate concentration with distilled water and filtered with a 0.45 μm Millipore filter to avoid contamination.

#### Field emission scanning electron microscopy (FE-SEM)

The GelMA nanoparticles were dispersed into water using stirring and sonication for 30 s. The dispersions were then applied on a clean glass slide, dried, and then vacuum-coated with gold. Digital images of the samples were acquired with a Hitachi S4160 field emission scanning electron microscope operating at 20 kV. Both scaffolds (*i.e.*, PolyHIPE–GelPEG and X-PolyHIPE–GelPEG) were vacuum-coated with gold and then imaged. The pore size distribution of the scaffold was obtained by the manual measurement of the pore size using JMicrovision 1.2.7 software followed by a Gaussian fitting to the data. For each sample, at least 100 pores were evaluated and sized. Also, 5 × 10^4^ cells were seeded on the scaffolds in a 24-well plate and incubated. 48 h after seeding, the scaffolds were taken out from wells and fixed with 70% ethanol. The scaffolds were placed on glass slides and then vacuum-coated with gold.

#### Thermogravimetric analysis (TGA)

The thermal properties and composition of the X-PolyHIPE–GelPEG–PCLs were investigated using a NETZSCH STA 409 PC/PG under a N_2_ flow of 20 ml min^−1^ with a heating rate of 10 °C min^−1^ from 30 to 600 °C.

## 3. Results and discussion

### 3.1. Preparation of GelMA and PEGDAC

Gelatin methacrylate (GelMA) was successfully prepared through methacrylic anhydride (MA) reaction with gelatin which was then confirmed by ^1^H NMR and FT-IR analysis. Since the amine groups of gelatins are amenable to react with MA, more than hydroxyl groups, to introduce methacrylamide groups on the gelatin chain, sometimes this product is called gelatin methacrylamide. Replacing amine groups with methacrylamide groups and introducing vinyl bonds on the gelatin were corroborated by ^1^H NMR and FT-IR (Fig. S1 and S2†).

**Fig. 1:**
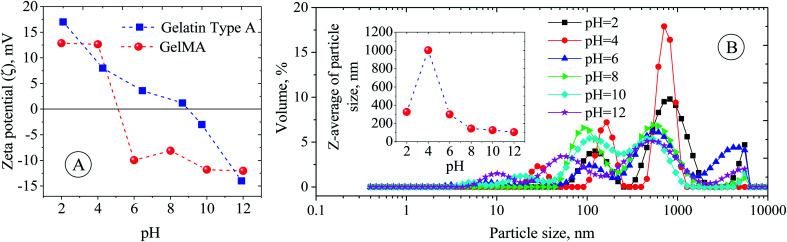
(A) Zeta (ζ) potential of gelatin and GelMA particles as a function of pH. (B) *Z*-Average hydrodynamic size and size distribution of GelMA particles as a function of pH.

**Fig. 2:**
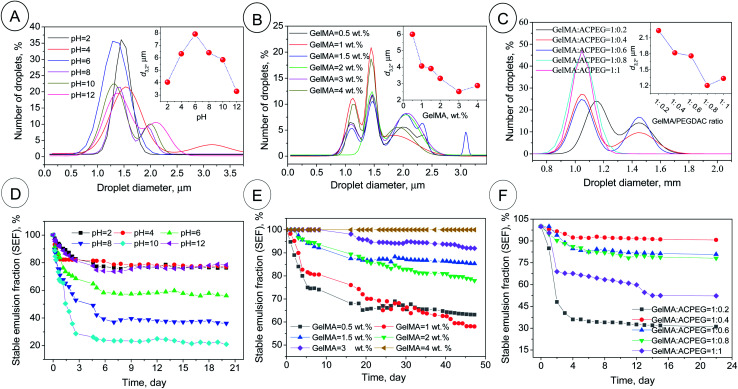
The droplet size distributions and *d*_3,2_ (inset figures) of the GelMA-stabilized coconut oil-in-water Pickering emulsions (A–C) along with the related emulsion stability in terms of stable emulsion fraction (SEF%) (D–F). PH series in Table 1; *φ*_oil_ = 74%, GelMA content = 1 wt%, GelMA/PEGDAC weight ratio = 10 : 1 and pH varied from 2 to 12 (A and D). GelMA series in Table 1; *φ*_oil_ = 74%, pH = 12, GelMA/PEGDAC weight ratio = 10 : 1, and GelMA content varied from 0.5 to 4 wt% (B and E). GelMA/PEGDAC series in Table 1; *φ*_oil_ = 74%, pH = 12, GelMA content = 4 wt%, GelMA/PEGDAC weight ratio varied from 10 : 2 to 10 : 10 (C and E). The data for size distribution are fitted by the Gaussian equation using Origin 2019b software.

The used gelatin type-A in this study was derived from pigskin, and an isoelectric point (IEP) of around 9 was found for it (Fig. 1A). However, the chemical modification of gelatin by MA has led to the shift of the IEP from 9 to around 5 (Fig. 1A). This shift in IEP makes the GelMA behave like gelatin type-B.^28^ The reason for the IEP shift most probably lies in reducing the number of protonable amine groups responsible for the positive charges on the gelatin chains. At pH < 5, the surface charge (zeta (ζ) potential) of the GelMA chain is positive due to the protonation of remaining amine groups, and it goes to higher positive values as the aquatic environment becomes more acidic, ζ-potential = ∼+12 mV at pH 2 (Fig. 1A). Also, at pH > 5, the ζ-potential is negative due to the deprotonation of ammonium and carboxylic groups of the GelMA, and it goes to higher negative values as the aquatic environment becomes more alkaline; ζ-potential = ∼−12 mV at pH 12 (Fig. 1A). Bearing in mind that gelatin and its derivatives (here GelMA) are amphiphilic biopolymers and can easily assemble into different kinds of aggregates under the defined processing conditions. Therefore, the pH variation changes the surface charge of GelMA and can impact the aggregation size of GelMA, namely GelMA nanoparticles (Fig. 1B). Because the GelMA chains like gelatin are soft and their swelling behavior and aggregation relate to the solvation degree of constitutive protein units and the balance of inter-/intrachain attractive and repulsive interactions. Although a minimum size of GelMA particles was expected around the IEP due to charge neutralization, an average size of around 1000 nm was found for it. It is believed that the numerical equilibrium of negative and positive charges on the surface of small nanoparticles increases their tendency to aggregate and produce large particles. However, the average particle size decreases dramatically on both sides of the IPE, so that it decreases to 320 nm at pH = 2 and 100–140 nm at pH = 8–12 (Fig. 2B). The enhanced negative and positive charges of GelMA nanoparticles, respectively, prevent particle–particle aggregation under the basic and acidic conditions, leading to a more stable colloidal dispersion. Fig. S3† shows a non-spherical morphology of GelMA particles (prepared at pH = 12) with a size of around 150 nm.

### 3.2. GelMA stabilized Pickering HIPEs

It is well known that gelatin is a denatured, biodegradable, and nonimmunogenic protein obtained by controlled hydrolysis of collagen's triple-helix structure into single-strain molecules. As mentioned above, gelatin as an amphiphilic biopolymer can easily assemble into different aggregates under the defined processing conditions like pH media. Therefore, the stabilization of emulsions based on the gelatin nanoparticles is due to viscoelastic shell formation around the droplets arresting the coalescence phenomenon.^32^ Besides, Pickering emulsions' fundamentals mention that narrowly distributed small particles have a higher capability to stabilize emulsion droplets due to their higher surface area, rearrangement, and packing ability.^33^ Also, particles with negative or positive surface charges can produce highly stable droplets because of the droplets' existing repulsion force with the same charge. These features avoid coalescence phenomena, leading to the smaller droplets.^33^ To examine the emulsifying potential of GelMA nanoparticles, coconut oil-in-water HIPEs were formulated, and affecting parameters on emulsion features (like pH, GelMA concentration, and GelMA/PEGDAC weight ratio) were investigated precisely. Powerful ultrasound waves were utilized for emulsion formation. Due to higher energy density input by ultrasound waves, any unwanted aggregations of GelMA nanoparticles in the continuous aquatic phase are broken down into smaller particles. This produces many effective particulate emulsion stabilizers that can adsorb immediately at the freshly created oil–water interfaces.^34,35^ Accordingly, too small droplets were found for all the prepared emulsions in this work.

#### The effect of pH on the emulsion features

The optical microscopy images of the as-prepared coconut oil-in-water emulsions as a function of pH (pH series in Table 1) are shown in Fig. S6.† The image evaluation revealed that the maximum droplet size (*d*_3,2_) of around 8 μm was obtained around pH = 6. But, the droplet size distribution and *d*_3,2_ were narrowed and decreased, respectively, by acidification and alkalifying. So, the *d*_3,2_ of 4 and 3.2 μm was calculated for pH = 2 and 12, respectively (Fig. 2A). This behavior was in correlation with the variation of ζ-potential and *Z*-average of GelMA particles with pH. At pH > 6 and pH < 6, the average size of GelMA nanoparticles was decreased due to the prevented particle–particle aggregation caused by the intensified repulsion forces between particles with the same surface charge (Fig. 1). Since the small particles lead to the small emulsion droplets and *vice versa*, the max *d*_3,2_ was obtained around the isoelectric point of GelMA at pH = 6, which has the largest GelMA particles (Fig. 1 and 2A). In contrast, the formed large number of small GelMA particles at both sides of pH = 6 led to the low values of *d*_3,2_. Moreover, the occurred limited coalescence phenomenon due to the droplets' repulsion forces with the same charge induced by negatively surface-charged GelMA particles (at pHs higher than 6) and positively surface-charged GelMA particles (at pHs lower than 6) led to the narrow droplet size distributions.^36^ The stability of emulsions was monitored for a month, and creaming was observed in all emulsions due to the lower density of coconut oil than water and imposition of buoyancy forces on coconut oil droplets. So, the smaller droplets migrate more slowly than the larger ones to the top of the vial. Consequently, the creaming occurs slowly for small droplets compared to larger ones.^37^ The highest stable emulsion fraction (SEF%) of around 80% at the steady-state was found for pH = 2, 4, and 12 (Fig. S9† and 2B). The main reason for these higher emulsion stabilities lies in the lower buoyancy force on the smaller droplets formed due to the occurred limited coalescence at these pHs. According to the uniformity of droplet size, obtained smallest *d*_3,2_ and highest emulsion stability, pH = 12 was selected as the optimum value for the following investigations.

#### The effect of GelMA content on the emulsion features

The optical microscopy images of the as-prepared coconut oil-in-water emulsions as a function of GelMA nanoparticles (0.5, 1, 1.5, 2, 3 and 4 wt%, GelMA content series in Table 1) at optimized pH = 12 are shown in Fig. S7.† The evaluation of images revealed that with GelMA content increasing in the continuous aquatic phase, the *d*_3,2_ and size distribution were decreased and narrowed, respectively (Fig. 2C). The partially covered droplets experience coalescence at lower nanoparticle contents to reduce the total interfacial area between water and oil, leading to larger droplets and higher *d*_3,2_ values, while the *d*_3,2_ decreases with GelMA content due to fully-covered droplets and prevented coalescence caused by the abundance of available nanoparticles (Fig. 2C and S7†). The other reason for produced small droplets is likely due to the enhanced viscosity of the continuous phase resulting from excess GelMA nanoparticles. The formed transient 3D networks through particle–particle interactions can increase the viscosity (*η*) and reduce droplets' diffusion coefficient (*D*_diff_). If Stokes' law is applicable here (*D*_diff_ = *k*_B_*T*/6π*ηr* in which *r* is the radius of droplets, *k*_B_ is the Boltzmann constant, and *T* is the absolute temperature), the resultant smaller *D*_diff_ caused by higher viscosity can decrease the collision frequency of droplets and therefore the rate of coalescence. All these can lead to smaller droplets. Moreover, this enhanced viscosity and the formed 3D network can entrap the droplets and impede droplet migration to the emulsion surface.^38^ Hence, the creaming phenomenon or, on the other hand, emulsion instability slows down. The highest emulsion stability was obtained at 4 wt% of GelMA content with an ESF of around 100% over 2 months, while the emulsion stability was suppressed with decreasing GelMA content (Fig. 2D). Due to the lower *d*_3,2_ value, uniformity in size distribution, and hence higher emulsion stability, the GelMA content of 4 wt% was selected as the optimum value for the subsequent investigations.

#### The effect of GelMA/PEGDAC weight ratio on the emulsion features

The optical microscopy images of the as-prepared coconut oil-in-water emulsions as a function of GelMA/PEGDAC weight ratio (1 : 0.2, 1 : 0.4, 1 : 0.6, 1 : 0.8, and 1 : 1, GelMA/PEGDAC series in Table 1) at optimized pH = 12 and constant GelMA content of 4 wt% are shown in Fig. S8.† The evaluation of images and corresponding statistical analysis of emulsion droplets revealed that the *d*_3,2_ and droplet size distribution decreased and narrowed with decreasing GelMA/PEGDAC ratio (or increasing PEGDAC content) (Fig. 2E). The main reason lies in the continuous aquatic phase's enhanced viscosity due to the increasing total content of polymers (GelMA and PEGDAC). As mentioned above, the improved viscosity can reduce the diffusion coefficient of droplets (*D*_diff_), diminishing the probability of droplet collision and coalescence phenomenon. Since the samples with GelMA/PEGDAC ratios of 1 : 0.4, 1 : 0.6, and 1 : 0.8 showed the highest emulsion stability (Fig. 2F), the ratio of 1 : 0.8 was chosen to prepare a soft and flexible porous scaffold.

### 3.3. Preparation of PolyHIPE–GelPEG with a semi BioSyn–IPN structure

**Scheme 1:**
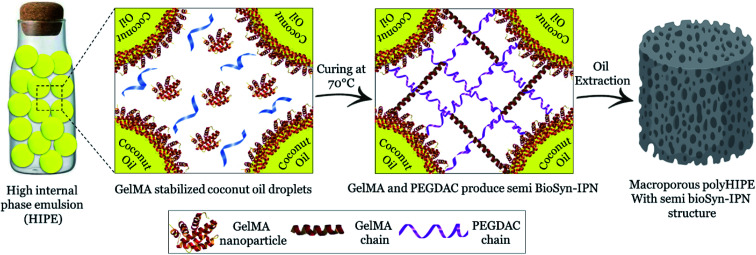
Schematic illustration for fabrication of PolyHIPE–GelPEG with a semi BioSyn–IPN structure.

**Fig. 3:**
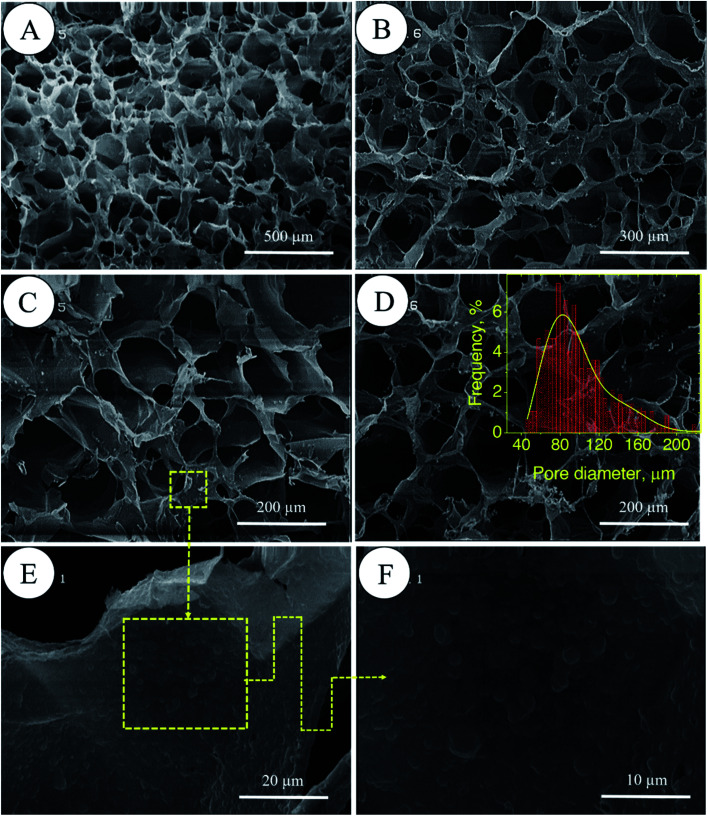
FE-SEM micrographs of PolyHIPE–GelPEG scaffolds without a crosslinker at different magnifications (A–D). The magnification of the inner surface of pores confirms the presence of GelMA nanoparticles (E and F). The pore size distribution of the scaffold was manually obtained by the manual measurement of pore size using JMicrovision 1.2.7 software; the solid curve is a Gaussian fit to the data.

**Fig. 4:**
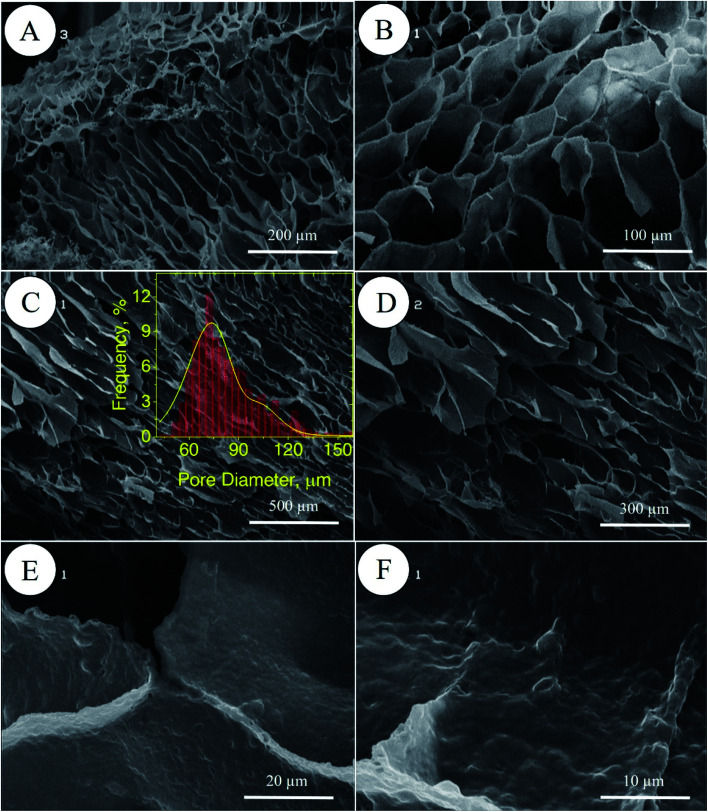
FE-SEM micrographs of the X-PolyHIPE–GelPEG scaffold (crosslinked by ethylene glycol dimethacrylate) at different magnifications. Surface morphology (A and B) and cross-sectional area of the scaffold (C and D). The observed roughness inside the pores comes from the GelMA nanoparticles used for particulate stabilization of the emulsion nanoparticles (E and F). The pore size distribution of the scaffold was manually obtained by the manual measurement of pore size using JMicrovision 1.2.7 software; the solid curve is a Gaussian fit to the data.

In porous polyHIPE preparation, the monomers or macromonomers are polymerized in the continuous phase followed by internal phase extraction. Herein, the polymerization of GelMA and PEGDAC macromonomers occurs in the aquatic phase of Pickering high internal phase of the coconut oil-in-water emulsion stabilized by GelMA nanoparticles. Then, the coconut oil and unreacted macromonomers were extracted using toluene and water, respectively, and finally, a porous PolyHIPE–GelPEG hydrogel with a semi BioSyn–IPN structure was obtained (Scheme 1 and Fig. S10A†). Based on the systematic evaluation of the effect of different parameters on the emulsion features, the following formulation was found suitable for the HIPE emulsion and therefore PolyHIPE–GelPEG preparation: GelMA = 4 wt% based on the aquatic phase, pH = 12, *φ*_oil_ = 74%, and a weight ratio of GelMA/PEGDAC = 1/0.8. During the polymerization of macromonomers, the polymer layers around droplets experience a significant shrinkage, and hence pores and small voids inside pores are formed to produce interconnected porous structures. These pores and voids can even be enlarged to promote porosity during the post-heating and solvent washing process, *i.e.*, internal phase extraction and purification. The FE-SEM images unfolded a uniform porous morphology for the prepared PolyHIPE–GelPEG with narrowly distributed pore sizes ranging from 60 to 180 μm (Fig. 3A–D). The observed roughness in the pores' inner wall is because of GelMA nanoparticles used as emulsion particulate-stabilizers (Fig. 3E and F). Apart from the stabilizing role of GelMA nanoparticles, they can take part in the curing process and be a part of the final scaffold's integrated structure. Furthermore, the nanoscale surface roughness assists cellular adhesion and proliferation. To improve the prepared scaffold's dimensional stability, ethylene glycol dimethacrylate (EGDMA), as a crosslinker, was added in different contents (0.05, 0.11, and 0.15 mmol) to the emulsion formulation and then polymerized. After removing the inner phase, an integrated porous polymer structure with appropriate mechanical properties was obtained, called X-PolyHIPE–GelMA/PEG (Fig. S10B†). This structure was very flexible and easily bent in the wet state without breaking or deformation. Moreover, the FE-SEM images revealed a porous structure with uniformly distributed pores ranging from 50 to 100 μm (Fig. 4). In addition, the SEM images revealed that the pores were connected through the cavities. The size of pores and cavities can be predictably increased multiply in the swelled state. The pore interconnectivity or in other words open-cell structure is crucial for oxygen and nutrient transportation to the cells, and removal of waste products from the scaffolds.^21^ Also, this interconnectivity provides a suitable pathway for cell migration inside the scaffold for uniform cell growth throughout the scaffold. Obviously, the pores of X-PolyHIPE–GelPEG are smaller than those of PolyHIPE–GelPEG, resulting from induced shrinkage by EGDMA crosslinker. Altogether, the surface roughness, suitable flexibility, and porosity features make this scaffold a candidate for skin tissue engineering applications, in which the elasticity comes from flexible PEG segments,^39^ and hydrogel properties come from both PEG and gelatin segments.

### 3.4. Preparation of final scaffolds X-PolyHIPE–GelPEG–PCL

Poly(ε-caprolactone) (PCL), as a semi-crystalline aliphatic polyester, has excellent biocompatibility, biodegradability, and easy processability (melting point at 60 °C), making it an interesting substrate for tissue engineering. However, like other synthetic polymers, PCL also lacks surface wettability and surface functional groups to improve cell attachment, which is a crucial parameter in tissue engineering. So, the concepts of hybrid scaffolds have been introduced to tackle this limitation. A combination of natural biopolymers and synthetic polymers is an acceptable way to design and engineer hybrid scaffolds. Although the natural biopolymers (such as collagen, gelatin, fibronectin, laminin, alginic acid, and chitosan) promote the extracellular matrix and cell adhesion, the related scaffolds are mechanically weak. The hybridization of synthetic polymers with natural polymers supports the final scaffold's dimensional stability and cell growth simultaneously. Moreover, it has been proven that the scaffold's surface wettability is vital for cell proliferation because the cells require a balanced hydrophobic–hydrophilic microenvironment to attach, stretch and grow well.^40^ Accordingly, in the present study, the final scaffolds were fabricated by incorporating PCL into the porous structure of X-PolyHIPE–GelPEG scaffolds *via* the infiltration method using the PCL solutions in different contents; 0, 2.5, 5, 7.5 and 10 wt%, respectively, called X-PolyHIPE–GelMAPEG–PCL-0, X-PolyHIPE–GelMAPEG–PCL-2.5, X-PolyHIPE–GelMAPEG–PCL-5, X-PolyHIPE–GelMAPEG–PCL-7.5, and X-PolyHIPE–GelMAPEG–PCL-10.

#### Characterization of the prepared scaffolds

**Fig. 5:**
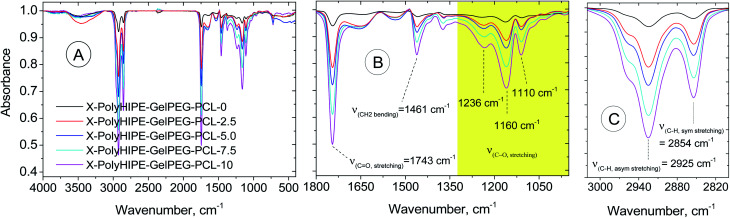
FT-IR spectra of a bare scaffold (X-PolyHIPE–GelPEG–PCL-0) and PCL incorporated scaffolds (X-PolyHIPE–GelPEG–PCLs).

FT-IR spectra were obtained to demonstrate the composition of the final scaffolds (Fig. 5). Compared with the X-PolyHIPE–GelPEG scaffold, the intensity of whole vibration peaks related to the PCL segments of the final scaffolds (*e.g.*, X-PolyHIPE–GelPEG–PCL) increased with the increase of PCL content. These peaks include symmetric and asymmetric stretching vibration of aliphatic C–H (2854 and 2925 cm^−1^, respectively), C–O stretching (1110, 1160, and 1236 cm^−1^), CH_2_ bending (1461 cm^−1^), and C=O stretching (1743 cm^−1^).

**Fig. 6:**
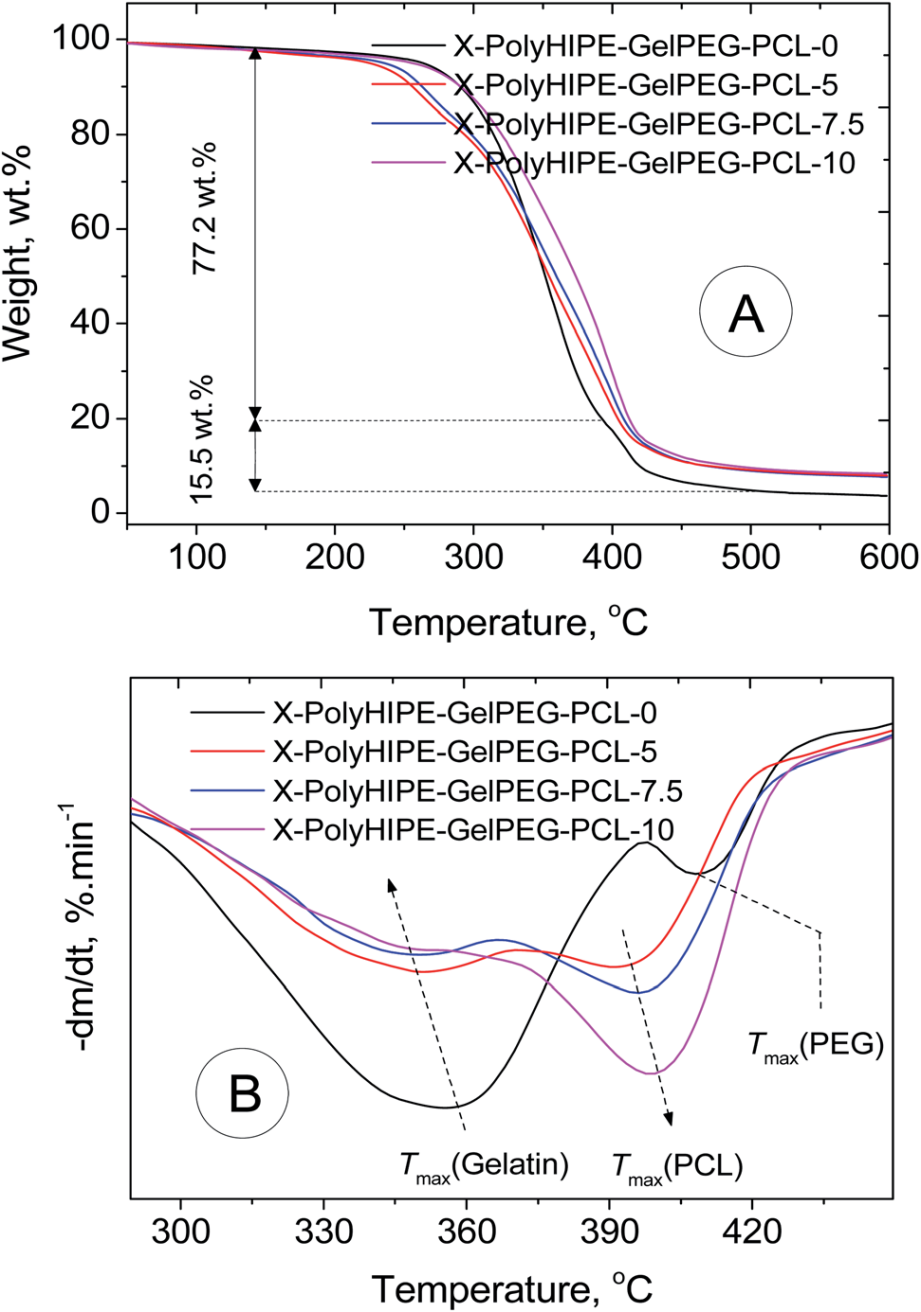
TGA (A) and DTG (B) thermograms of a bare scaffold (X-PolyHIPE–GelPEG–PCL-0) and PCL incorporated scaffolds (X-PolyHIPE–GelPEG–PCLs).

TGA/DTG analysis was performed to determine the final scaffolds' composition and thermal characteristics (Fig. 6). In the scaffold without any PCL content (*e.g.*, X-PolyHIPE–GelPEG–PCL-0), a weight loss of 7.28 wt% in the temperature range 50–150 °C relates to the removal of solvent impurities and physisorbed water. Also, a weight loss of 77.20 wt% in the range of 150–400 °C relates to the destruction of the gelatin skeleton, and a weight loss of 15.52 wt% is associated with the PEG degradation. The detailed investigation unfolded that the PCL content increased with increasing PCL concentration of solutions used in the infiltration method, while the PEG and gel contents decreased simultaneously. Interestingly, the temperature at the maximum degradation rate (*T*_max_) of gel decreased with increasing PCL content (Fig. 6 and Table 2). During the infiltration method, the PCL chains can gradually diffuse into the porous scaffolds and settle down on the pores' inner wall. Accordingly, it's believed that the heat transfer was intensified with PCL content because of the high heat transfer coefficient of PCL (0.3 W (m^−2^ K^−1^)) compared to gelatin (0.19 W (m^−2^ K^−1^)).^41^ Also, the *T*_max_ of PCL increased with PCL content (Fig. 6 and Table 2). Although the PCL chain is surface adsorbed on the pores' inner wall at low PCL content, there are no favorable interactions between the PCL and gelatin chains due to their different nature of hydrophobicity. And due to the fact of weak interactions, the low *T*_max_ was observed for the PCL. And in high PCL content, due to the thickening of the PCL layer on the pore wall surface and the abundance of PCL chains, they tend to crystallize. Therefore, this might lead to increasing *T*_max_ of PCL, getting closer to the value reported for pure PCL (405 °C) (Table 2).^42^

**Table 2:** Thermal characteristics and determined composition of a bare scaffold (X-PolyHIPE–GelPEG–PCL-0) and PCL incorporated scaffolds (X-PolyHIPE–GelPEG–PCLs)

Sample	*T*_max_ (gel), °C	*T*_max_ (PEG), °C	*T*_max_ (PCL), °C	Gel content, wt%	PEG content, wt%	PCL content, wt%
X-PolyHIPE–GelPEG–PCL-0	358	408	—	77.2	15.5	—
X-PolyHIPE–GelPEG–PCL-5	352	n/a	394	58.1	11.7	18.5
X-PolyHIPE–GelPEG–PCL-7.5	348	n/a	397	51.2	10.3	27.2
X-PolyHIPE–GelPEG–PCL-10	345	n/a	399	43.7	8.8	35.6

### 3.5. Cell culture

**Fig. 7:**
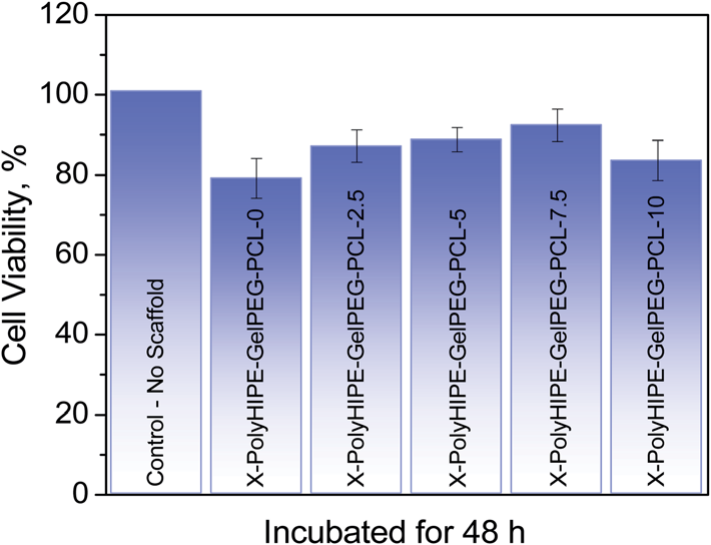
Cell viability of mouse fibroblast cells (L929) grown on the PolyHIPE–GelMA–PCL scaffolds containing different weight percent of PCL incubated for 48 h.

The application potential of the prepared scaffolds for tissue engineering was evaluated. In this regard, the adhesion of L929 cells (mouse fibroblast cells) on the X-PolyHIPE–GelMA–PCLs after 48 h of incubation was assessed by MTT assay, and the corresponding cell morphology was investigated by FE-SEM images. Overall, the scaffolds were an appropriate substrate for cell growth even one contains no PCL (Fig. 7). But the highest cell growth was found for X-PolyHIPE–GelMA–PCL-7.5. It seems that in this scaffold, there is a proper balance between hydrophobicity and hydrophilicity, providing a suitable microenvironment for cell adhesion and growth.^43^ Probably at the low PCL contents, the surface of pores existing in the scaffolds had been partially covered by PCL chains, leading to a more hydrophilic surface for cell growth. And at the higher PCL contents, the surface of pores had been covered with thick PCL layers, providing a highly hydrophobic surface for cell growth. Therefore, the fibroblast cells have not been grown enough like X-PolyHIPE–GelMA–PCL-7.5 due to the lack of proper balance between hydrophilicity and hydrophobicity.

**Fig. 8:**
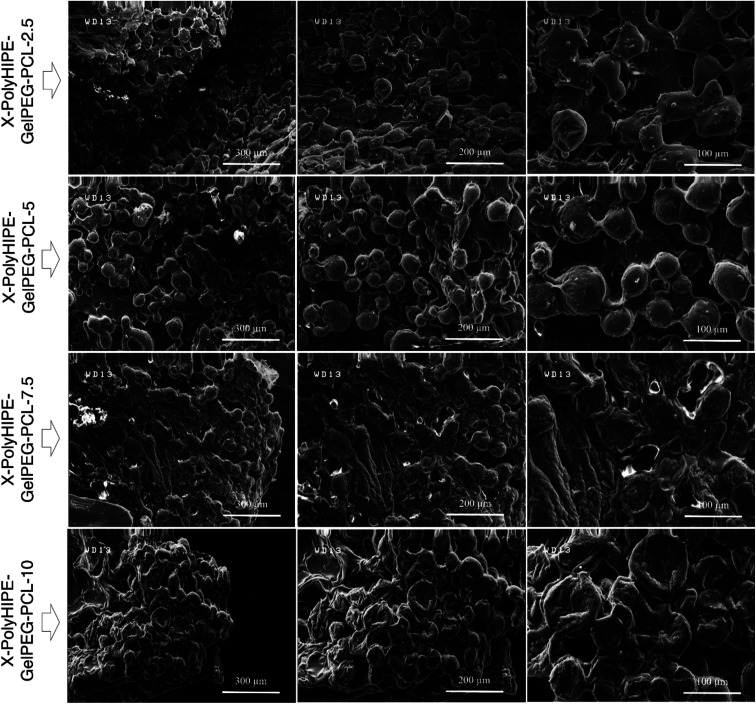
FE-SEM micrographs of mouse fibroblast cells (L929) cultured on the X-PolyHIPE–GelPEG–PCL scaffolds after 48 h of incubation.

Moreover, at higher PCL content (*i.e.*, X-PolyHIPE–GelPEG–PCL-10), the pore size decreases because the thicker PCL layer may lead to tension between the cells and lack of nutrients for cell growth. The FE-SEM micrographs of the cultured mouse fibroblast cells (L929) on the scaffolds are shown in Fig. 8. The images revealed a spherical and polyhedral morphology for cells more like their normal phenotype.^43^

## 4. Conclusion

Gelatin methacrylate (GelMA) was successfully exploited as a particulate emulsifier for stabilizing high internal phase Pickering coconut oil-in-water emulsions. Copolymerization of existing poly(ethylene glycol) diacrylate (PEGDAC) and GelMA in the continuous aquatic phase of the emulsion followed by extraction of the internal phase led to a series of hierarchical macroporous 3D scaffolds with a semi-interpenetrating network (semi IPN) structure. Due to the interlacing of a biopolymer with a synthetic polymer, these structures had a semi BioSyn–IPN. Moreover, the existing GelMA nanoparticles at the surface of emulsion droplets could participate in the curing process and incorporate into the final structure. The effect of different parameters such as pH, GelMA content, and GelMA/PEGDAC weight ratio on the emulsion features (before polymerization) was systematically investigated. And the results showed that the GelMA particles were in their max size of around 1000 nm at pH 6 due to the isoelectric point of GelMA at the same pH. These large GelMA aggregates led to a large emulsion droplet with around 8 μm, while on both sides of pH 6, the GelMA particle size and emulsion droplets decreased gradually (up to 100–140 nm and 3–4 μm, respectively). This was because of the abundance of positive (at pH < 6) and negative charges (at pH > 6) on the GelMA nanoparticles and hence emulsion droplets. The prevented coalescence due to the same charge repulsion force was the main reason for this decrease. Moreover, the stability and emulsion droplet size were enhanced and increased, respectively, with GelMA content increasing and GelMA/PEGDAC weight ratio decreasing. These trends lie in the prevented coalescence phenomenon caused by the improved viscosity and likely partially formed network by GelMA chains in the continuous phase. Hence, the following formulation was selected for scaffold preparation: *φ*_oil_ = 74%, pH = 12, GeMA = 4 wt%, and GelMA/PEGDAC = 10/8. The obtained scaffold, named polyHIPE–GelPEG, had a uniform open-cell macroporous structure with a pore size in a range of 60–180 μm, while the corresponding crosslinked scaffolds with ethylene glycol dimethacrylate, named X-polyHIPE–GelPEG, had a small pore size in a range of 50–100 μm. The PCL chains in different contents were infiltrated into the scaffold to bring a balance of hydrophilicity and hydrophobicity to the scaffold. The biocompatibility test proved that the scaffold containing 51.2 wt% GelMA, 10.3 wt% PEG, and PCL 27.2 wt% provided a suitable microenvironment for mouse fibroblast cell (L929) growth with a normal morphology. These results show that this strategy can produce scaffolds with a great potential for tissue engineering applications.

## Conflicts of interest

There are no conflicts of interest to declare.

## Supplementary Material

RA-011-D1RA03333F-s001
