# Supplementary material for: Porous scaffolds with the structure of an interpenetrating polymer network made by gelatin methacrylated nanoparticle-stabilized high internal phase emulsion polymerization targeted for tissue engineering
Source: RSC Adv. 2021 Jun 25;11(37):22544–55. doi: 10.1039/d1ra03333f (PMC9034234; doi:10.1039/d1ra03333f)
Supplement: RA-011-D1RA03333F-s001 [file RA-011-D1RA03333F-s001.pdf]

Electronic Supplementary Material (ESI) for RSC Advances.
This journal is © The Royal Society of Chemistry 2021

Supporting Information

Porous Scaffold with Structure of Interpenetrating Polymer Network Made by Gelatin Methacrylated Nanoparticle-Stabilized High Internal Phase Emulsion Polymerization Targeted for Tissue Engineering

Atefeh Safaei-Yaraziz[a], Shiva Akbari-Birgani[b], Nasser Nikfarjam[a,*]

[a]Department of Chemistry, Institute for Advanced Studies in Basic Sciences (IASBS), Zanjan 45137-66731, Iran

[b]Department of Biological Sciences, Institute for Advanced Studies in Basic Sciences (IASBS), Zanjan 45137-66731, Iran

Macromonomers' characterization

The FT-IR spectrum of gelatin type A and gelatin methacrylated (GelMA) are shown in Figure S1. The following peaks are observed for both gelatin and GelMA, including 3420 cm^{-1} (overlapped stretching vibration of O-H and N-H), 2928 cm^{-1} (stretching vibration of aliphatic C-H), 1240 cm^{-1} (stretching vibration of C-N, and bending vibration of N-H). Besides these peaks, the appearance of new vibration peaks in the GelMA spectrum indicates successful synthesis of GelMA. These peaks include 3075 cm^{-1} (stretching vibration of =CH), 1620 cm^{-1} (stretching vibration of C=C), and 1645 cm^{-1} (stretching vibration of C=O).[1] ^{1}H NMR analysis also confirmed the synthesis of GelMA (Figure S2). Compared to the spectrum of gelatin, new peaks at 5.2-5.6 ppm and 1.7 ppm were attributed to alkene protons and methyl protons of methacrylate, respectively. The peak at 2.8 ppm was also disappeared in the GelMA, indicating that the amine groups of gelatins were reacted with methacrylic anhydride.[1]

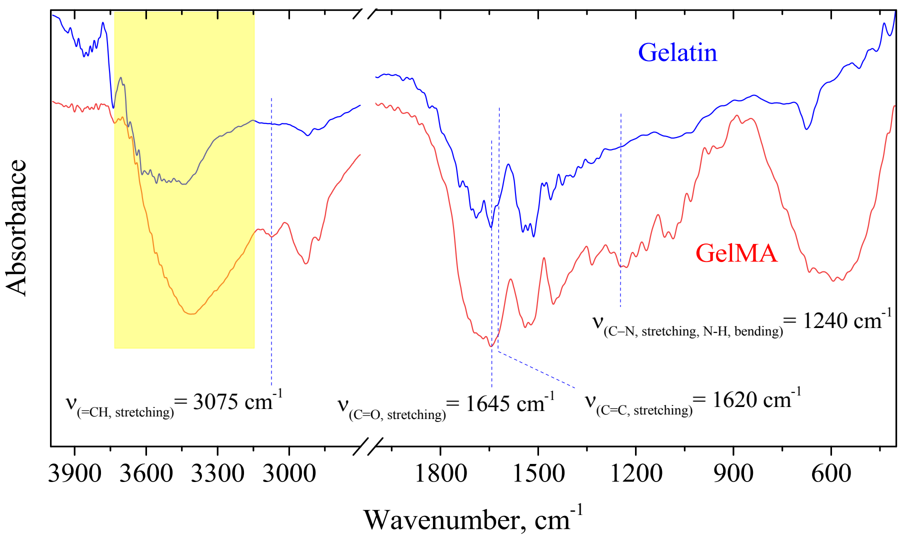

Figure S1. FT-IR spectrum of gelatin type A and GelMA.

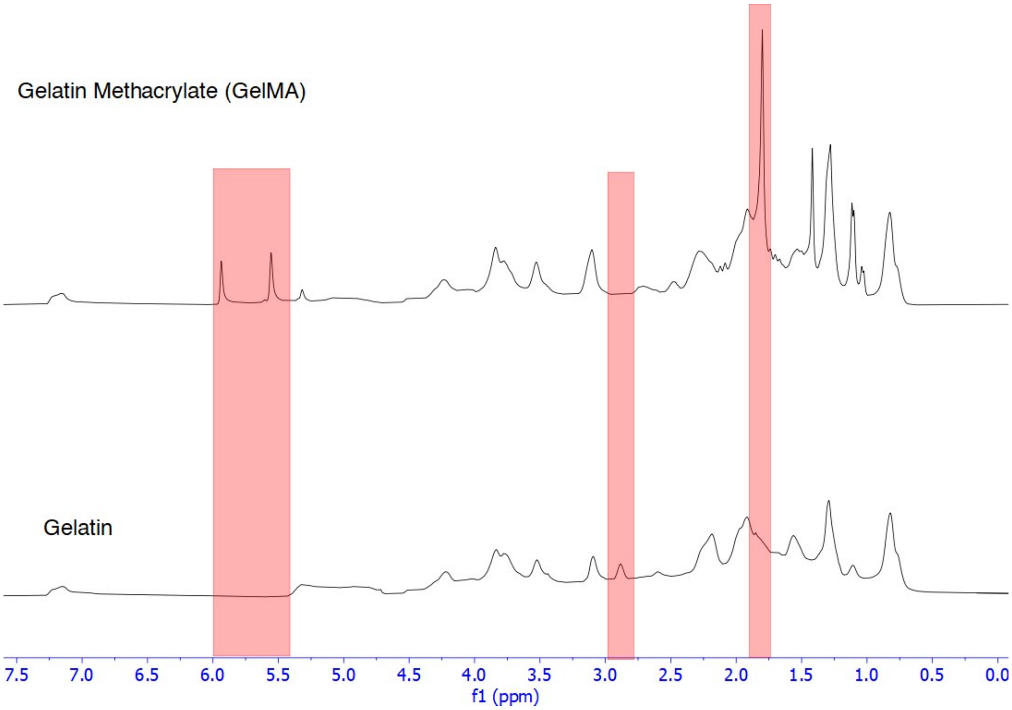

Figure S2. ^{1}H NMR spectrum of gelatin type A and GelMA in $CDCl_3$ solvent.

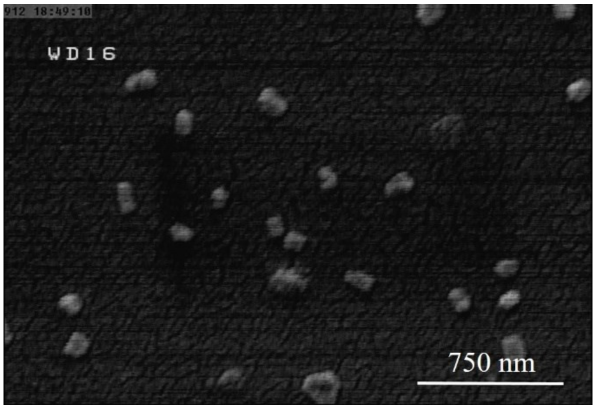

Figure S3. FE-SEM micrographs of GelMA nanoparticles obtained at pH=12 with a size average of around 150 nm.

The FT-IR spectrums of PEG and PEG diacrylate (PEGDAC) were shown in Figure S4. The observed peaks for PEG are including; 3200-3600 cm^{-1} (for stretching vibration of O-H), 2850-2950 cm^{-1} (for stretching vibration of C-H), 1006-1180 cm^{-1} (for stretching vibration of C-O), 1351 and 1457 cm^{-1} (for bending vibration of C-H). After the diacrylation of PEG, the stretching vibration of O-H was disappeared, and new prominent peaks appeared. All these new peaks are attributed to acrylate groups including 3091 cm^{-1} (for stretching vibration of =CH), 1731 cm^{-1} (for stretching vibration of C=O), 1535 cm^{-1} (for stretching vibration of C=C), and 748 cm^{-1} (for bending vibration of =CH). All these observations confirm the successful substitution of terminal hydroxyl groups of PEGs with acrylates. The presence of new peaks attributed to the alkenyl protons in PEGDAC in ^{1}H NMR analysis further confirmed the diacrylation of PEG (Figure S5).[2]

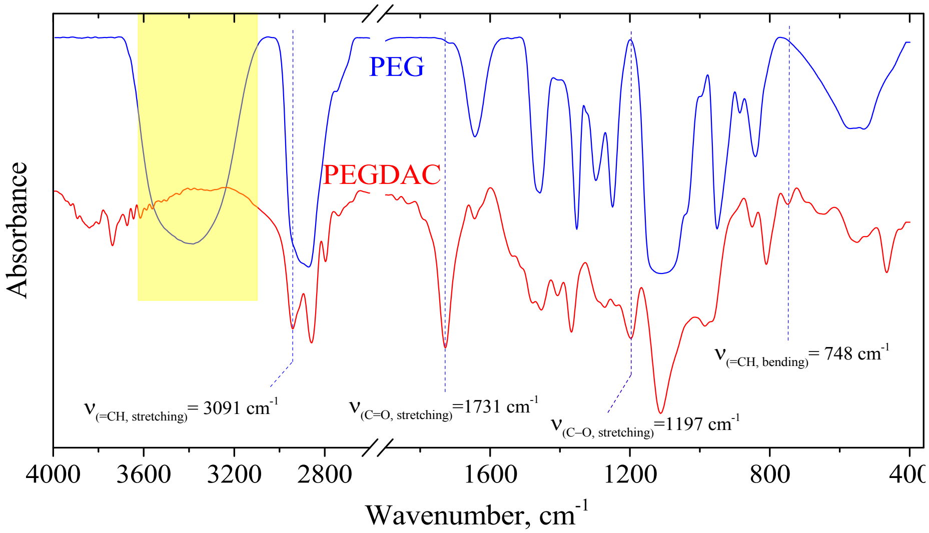

Figure S4. FTIR spectrum of PEG and PEGDAC.

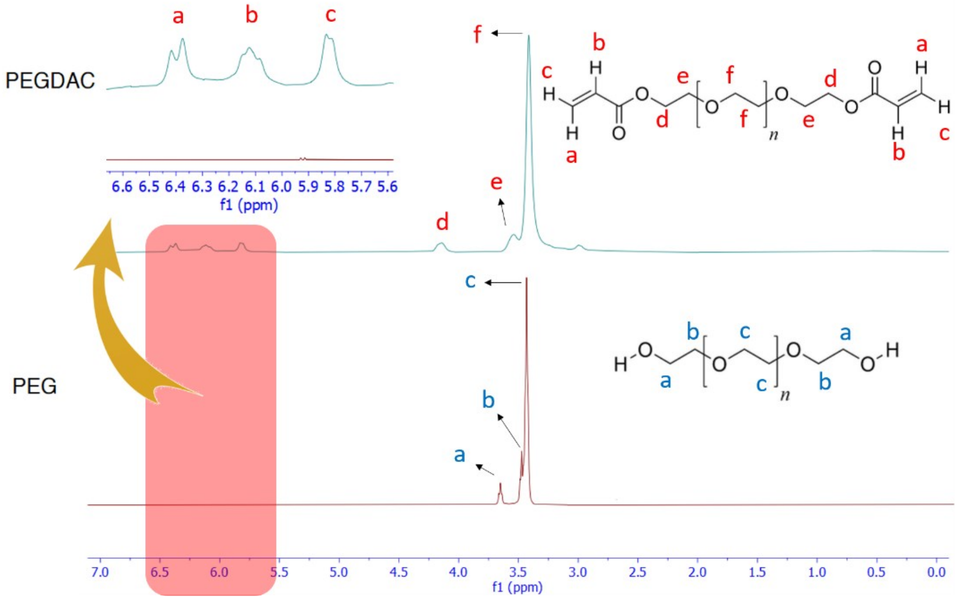

Figure S5. ^{1}H NMR spectrums of PEG and PEGDAC in $CDCl_3$ solvent.

Emulsion investigation

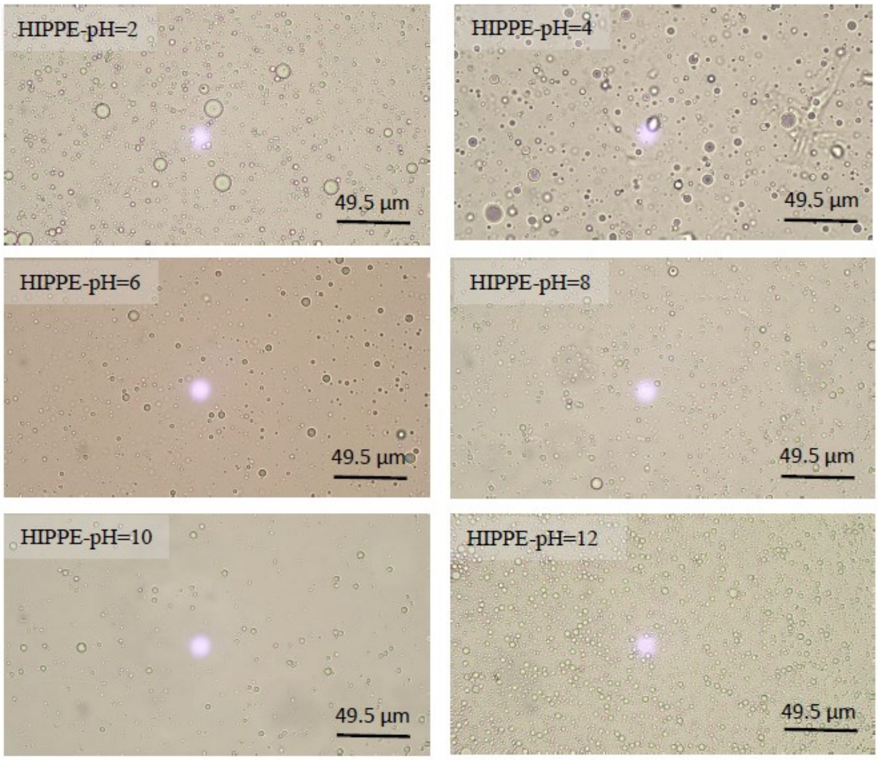

Figure S6. The pH effect on the features of prepared Pickering high internal phase emulsion (HIPE) by GelMA nanoparticles. PH series in Table 1; φ_{oil} = 74%, GelMA content = 1 wt.%, GelMA/PEGDAC weight ratio=10:1 and pH varied from 2 to 12.

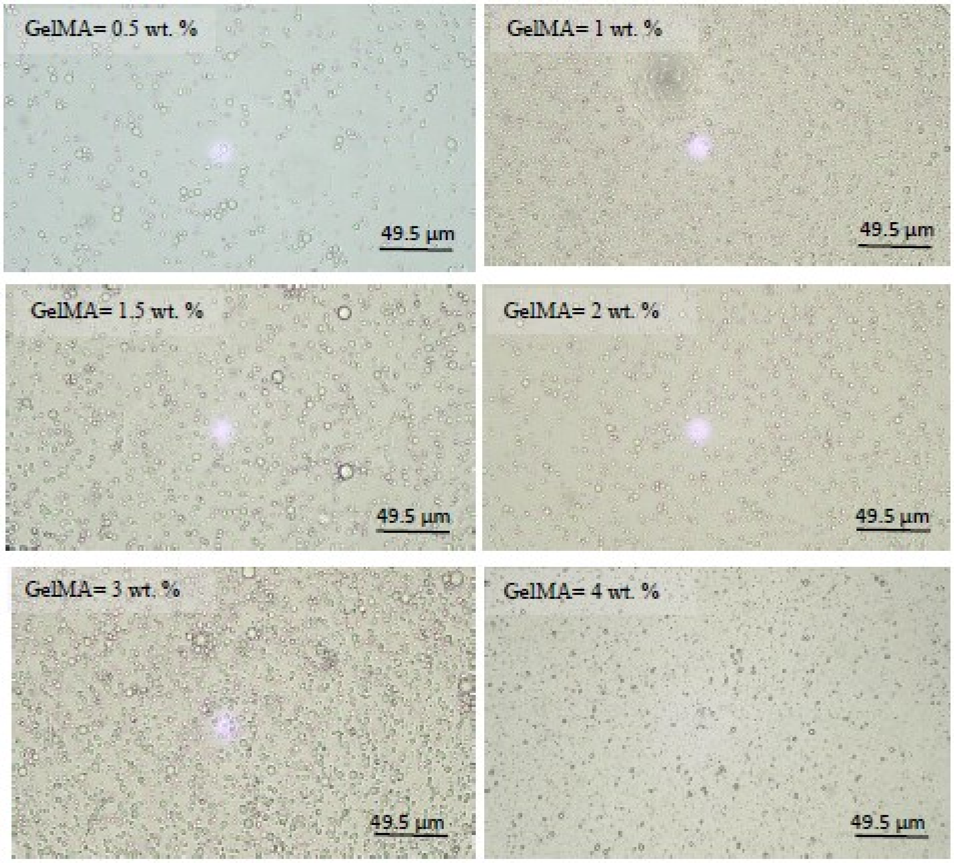

Figure S7. The GelMA nanoparticle content effect on the features of prepared Pickering high internal phase emulsion (HIPE) by GelMA nanoparticles. GelMA series in Table 1; φ_{oil} = 74%, pH = 12, GelMA/PEGDAC weight ratio=10:1, GelMA content varied from 0.5 to 4 wt.%.

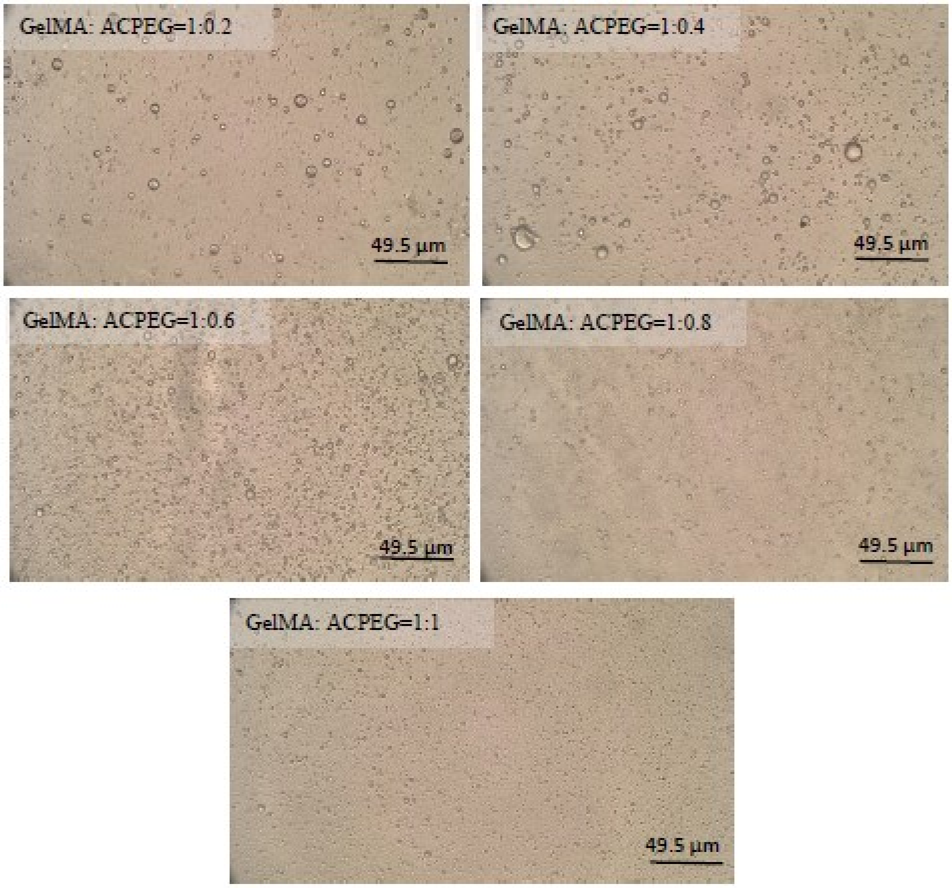

Figure S8. The effect of GelMA/PEGDAC weight ratio in the aquatic continuous phase on the features of prepared Pickering high internal phase emulsion (HIPE) by GelMA nanoparticles. GelMA/PEGDAC series in Table 1; φ_{oil} = 74%, pH=12, GelMA content = 4 wt.%, GelMA/PEGDAC weight ratio varied from 10:1 to 10:10.

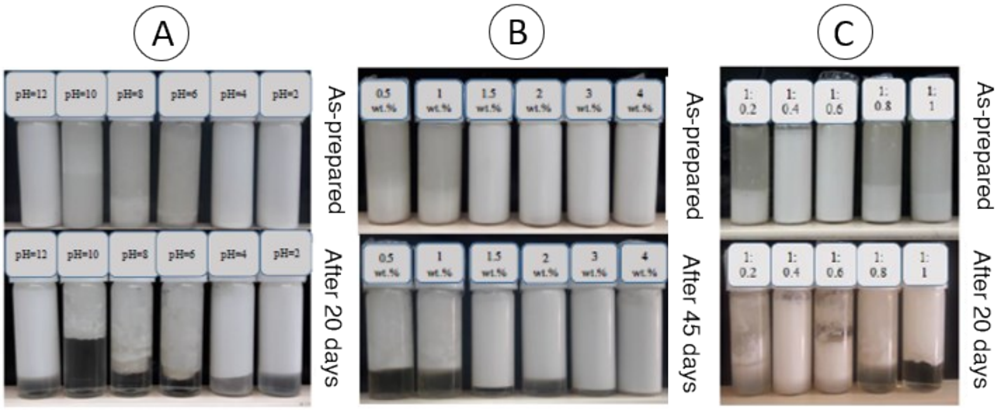

Figure S9. Emulsion stability of Pickering high internal phase emulsion stabilized by GelMA nanoparticles. A) pH series, B) GelMA nanoparticle series and C) GelMA/PEGDAC ratio series, see Table 1.

Scaffold characterization

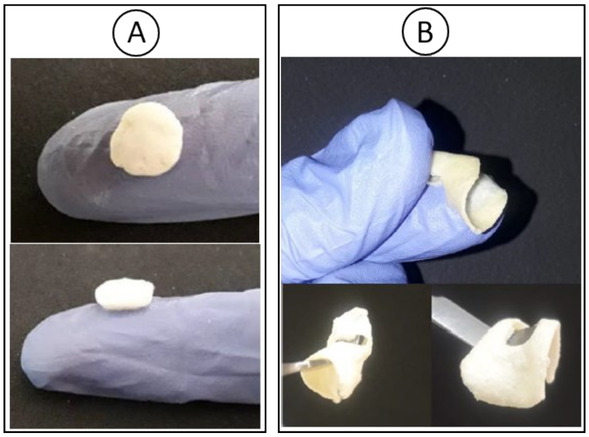

Figure S10. Digital photo of prepared PolyHIPE-GelPEG (A) and X-PolyHIPE-GelPEG (B).

Appendix

The below code is the coding in MATLAB software to recognize spherical emulsion droplets and measure their size to obtain droplet size distribution and mean diameter of droplets ($d_{3,2}$).

```
%insert parameters below:
%1) pixelSize=the size of each pixel on image
%2) rmin , rmax (rmax should be maximum 3 times larger than rmin),
%3) bright or dark,
%4) EdgeThreshold number and Sensitivity number (the last two one between 0
and 1)
%5) N=number of images
%color of circles in image are refer to:
%blue for first part
%green for second part
%red for third part
%black for fourth part
%pixel size:4x=2.032 10x=0.841 40x=0.214 100x=0.081 Micron
clc
clear all
close all
cd();
PixelSize=0.214;%pixelSize= the size of each pixel on image
N=6;% number of images
number=1
pathname=uigetdir();%path of folder cotaining images
for i=1:N
str= ['a (', num2str(i),')'];
nn=[pathname, '\',str,'.tif'];
a1=im2double(rgb2gray((imread(nn))));
a1=a1-min(a1(:));
```

```
a1=a1/max(a1(:));
b1=im2double(imbinarize(a1,0.87));
c=b1;
figure(i);imagesc(b1);
%% part1
[centers1, radii1] = imfindcircles(c,[2
6],'ObjectPolarity','bright','EdgeThreshold',0.1,'Sensitivity',0.895);
hold on
viscircles(centers1, radii1,'EdgeColor','b');
radii1=radii1';
%% part2
[centers2, radii2] = imfindcircles(c,[7
12],'ObjectPolarity','bright','EdgeThreshold',0.05,'Sensitivity',0.93);
hold on
viscircles(centers2, radii2,'EdgeColor','g');
radii2=radii2';
%% part3
[centers3, radii3] = imfindcircles(c,[13
40],'ObjectPolarity','bright','EdgeThreshold',0.07,'Sensitivity',0.9);
hold on
viscircles(centers3, radii3,'EdgeColor','r');
radii3=radii3';
%% part4
[centers4, radii4] = imfindcircles(c,[41
170],'ObjectPolarity','bright','EdgeThreshold',0.06,'Sensitivity',0.96);
hold on
viscircles(centers4, radii4,'EdgeColor','k');
radii4=radii4';
%%

radii=0;
ss1=isempty(radii1);
if ss1==0
    radii=radii1;
end

l=length (radii);
ss2=isempty(radii2);
if ss2==0
    x=length(radii2);
    Radii=zeros(1,l+x);
    Radii(1:l)=radii;
    Radii(l+1:l+x)=radii2;
    radii=Radii;
end

l=length (radii);
ss3=isempty(radii3);
if ss3==0
    x=length(radii3);
    Radii=zeros(1,l+x);
    Radii(1:l)=radii;
    Radii(l+1:l+x)=radii3;
    radii=Radii;
end
```

```
l=length (radii);
ss4=isempty(radii4);
if ss4==0
    x=length(radii4);
    Radii=zeros(1,l+x);
    Radii(1:l)=radii;
    Radii(l+1:l+x)=radii4;
    radii=Radii;
end

radii=radii*2*PixelSize;

if i==1
    Diameter=radii;

else
  xx=length(radii);
   ll=length(Diameter);
    diameter=zeros(1,ll+xx);
    diameter(1:ll)=Diameter;
    diameter(ll+1:ll+xx)=radii;
    Diameter=diameter;
end
% eval(['RadiiT' num2str(i) '=i'])=sort(radii);
% str2=['diameter of particles (',num2str(i),')'];
% nn2=[pathname,'\',str2,'xlsx'];
% xlswrite(nn2,(eval(['RadiiT' num2str(i) '=i']))')
% pause(2)
% number =number
radii=sort(radii);
radiiT=radii';

% xlswrite(nn2,radiiT')
pause(2)
number =number;
if i==1;
    RadiiTotal= vertcat(radiiT);
else
    RadiiTotal=vertcat(RadiiTotal,radiiT);
end
end
str2=['D_Total'];
 nn2=[pathname,'\',str2,'xlsx'];
xlswrite(nn2,RadiiTotal);


figure(i+1);h =
histogram(Diameter,'Normalization','probability');xlabel('Diameter of
particles(Micro meter)');ylabel('Number of particles (Normalized to 1)');

display('mean=')
MEAN=mean(Diameter,2);
display(MEAN)
```

References

[1] C. Li, C. Mu, W. Lin, *RSC Adv.* **2016**, *6*, 43663.

[2] K. H. Son, J. W. Lee, *Mater.* **2016**, *9*, DOI 10.3390/ma9100854.
